# Editorial: Molecular mechanisms of epithelial-mesenchymal transition in cancer metastasis

**DOI:** 10.3389/fonc.2022.1088205

**Published:** 2022-12-06

**Authors:** Amr Amin, Mariam Alyahyaee, Yingqiu Xie, Lubna Tahtamouni

**Affiliations:** ^1^ Biology Department, United Arab Emirates (UAE) University, Al Ain, United Arab Emirates; ^2^ Department of Biology, School of Sciences and Humanities, Nazarbayev University, Astana, Kazakhstan; ^3^ Department of Biology and Biotechnology, Faculty of Science, The Hashemite University, Zarqa, Jordan; ^4^ Department of Biochemistry and Molecular Biology, College of Natural Sciences, Colorado State University, Fort Collins, CO, United States

**Keywords:** epithelial-mesenchymal transition, therapy, cancer, EMT, ECM

Most malignant tumors are epithelial in origin. Epithelial cells are closely connected to one another through cell-cell interactions. These interactions rigorously determine where epithelial cells reside in the body and appear to be at odds with the ability of many malignancies to spread to other parts of the body (i.e., ability to metastasize). Almost 90% of cancer-related deaths are due to metastasis. Epithelial-mesenchymal transition (EMT), which occurs when epithelial cells change into mesenchymal cells with high mobility and migratory ability, provides the basis for tumor dissemination. Several studies have shown that EMT allows cells of solid tumors to become more malignant by increasing their invasiveness and metastatic activity. In this special issue on Epithelial-Mesenchymal Transition (EMT) as a Therapeutic Target in Cancer we have invited a few papers that address the role of EMT in metastasis and different possibilities to target EMT as potential therapeutic targets.

As an important regulator of transcription of many genes, the role of AF4/FMR2 family member 4 (AFF4) in colorectal cancer (CRC) was investigated in the first study of this Research Topic (Fang et al.) In their article, Fang et al. showed that downregulation of AFF4 in CRC patients is associated with poor prognosis. Authors also reported that AFF4 deficiency promotes cancer cell invasion and enhances the metastasis of CRC both *in vitro* and *in vivo*. Interestingly, suppression of EMT is shown to be associated with AFF4-induced transcription of CDH1 gene that encodes E-cadherin. Fang et al. then argued that AFF4 may potentially serve as an effective therapeutic target for CRC patients

In another investigation, Feng et al. developed and validated an EMT-related gene prognostic index (EMTGPI) based on only two genes (SFRP4 and SPP1) predicting biochemical recurrence (BCR) and drug resistance in prostate cancer (PCa) patients undergoing radical prostatectomy or radiotherapy. Given the high association of EMTGPI with many immune checkpoints including, but not limited to, M1 macrophages, authors proposed the presence of immune evasion in the progression of PCa.

Cytokines are a cornerstone of inflammation, a hallmark of hepatocellular carcinoma (HCC). In addition to its documented effect on EMT in cervical and breast cancer cells, Srivastava et al. demonstrated that, in HCC, IL-6 cytokine alone induces proliferation through modulating both STAT-3 and NFkB whereas TGF-β is found to induce EMT in liver cancer cells treated with such cytokine alone. Interestingly, simultaneous treatment with IL-6 and TGF-β shows hardly any effect on EMT markers. Authors reported that TGF-β attenuated IL-6-induced effects by limiting the receptor’s transcription level and modulating downstream signaling components and chromatin remodeling. Insights into alternative approaches to manipulate cytokine balance, according to researchers, can be developed into a future therapeutic strategy to treat HCC.

In many tumors, the expression of G-protein coupled receptor 56 (GPR56) has been shown to affect radio resistance and EMT. Ganesh et al. concluded that GPR56 is essential for the invasion, migration, and mesenchymal transformation of glioblastoma (GBM) cells. In their investigation, they showed that GBM cells had an enriched gene signature, pathway, and phosphorylation of proteins that may be related to the mesenchymal transition. Furthermore, GPR56 knockdown in GBM cells also exhibited increased cell invasion and migratory behavior. In addition, they found that transglutaminase 2 (TG2), a recognized GPR56 interactor, was likewise expressed at higher levels in the knockdown cells. Thus, Ganesh et al. hypothesized a possible molecular connection between the hypoxic niche, the regulation of the mesenchymal transition in GBM, and the inverse expression of GPR56 and TG2.

Extracellular Matrix Protein 1 (ECM1) is reported to contribute heavily to the progression of metastatic cancers and consequently to EMT process. In their article, Long et al. revealed that ECM1 overexpression can enhance EMT induction and CRC tumor progressions, and it correlates with tumor size and lymph node status in CRC patients. In their findings, the authors showed that ECM1 controls CRC metastasis and EMT processes *via* PI3K/AKT/GSK3B/Snail signaling axis and has an oncogenic influence on CRC formation and progression. In addition to CRC tissues, CRC cell lines which were assessed in this study were also reported to have an elevated ECM1 expression. Thus, Long et al. concluded that ECM1 represents a promising prospective biomarker for CRC that could serve as a viable therapeutic target especially for patients with metastatic CRC.

As the most abundant RNA binding proteins in the hnRNPA1s family, hnRNPA1 is known to regulate EMT transition and tumor metastasis *via* a number of different molecular processes. Han et al. showed that downregulation of hnRNPA1 induces migration, invasion, and EMT transition in lung cancer cells. They also demonstrated that hnRNPA1 prevents EMT transition *via* acting as an alternative splicing regulator and can indirectly influence tumor metastasis and EMT through controlling the alternative splicing of the LAS1L pre-mRNA. Biological effects of hnRNPA1 could offer a valuable foundation for future lung cancer research, authors concluded.

Finally, Gelissen and Huang elegantly offered an overview of intersections of endocrine pathways and EMT in endometrial cancer. They highlighted the limitation of the *in vitro* analyses that most studies on endometrial cancer cell lines employ. Authors then argued that *in vivo* studies using xenograft and transgenic models of endometrial cancer are required to validate the results seen in cell lines and recommended that future research should be focused on practical applications that could change the course of endocrine-mediated EMT in endometrial cancer. Singling out the improved outcomes of data linking endometrial cancer with metformin treatment and how that would support the role of multiple endocrinological disturbances in the EMT of endometrial cancer was Gelissen and Huang take-home message.

## Author contributions

MA and AA wrote the first draft. YX and LT revised the manuscript. All authors, finally, proofread and approved it.

